# Two new CRISPR-generated alleles in the *C. elegans mir-1022* gene.

**DOI:** 10.17912/micropub.biology.000233

**Published:** 2020-04-01

**Authors:** Connor Horn, Robert Sholl, Dustin Haskell, Shilpa Hebbar, Anna Zinovyeva

**Affiliations:** 1 Division of Biology, Kansas State University, Manhattan, KS

**Figure 1:**
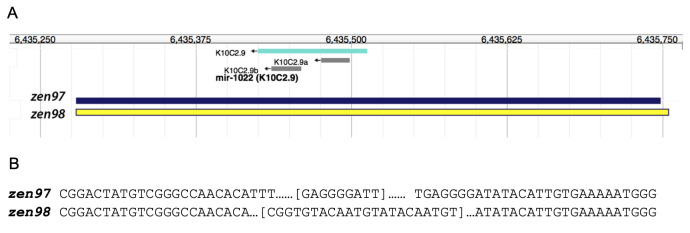
Using CRISPR/Cas9, two *mir-1022* deletions/insertions (indels), *zen97* and *zen98,* were generated. (A) A schematic of the *C. elegans mir-1022* locus. The location of *zen97* and *zen98* deletions are designated by the blue and yellow rectangle, respectively. (B) The genetic sequence of the *zen97* and *zen98* deletions’ endpoints in the *mir-1022* locus, with the deletions represented by the ellipsis. Insertion sequence within each allele is in brackets.

## Description

MicroRNAs (miRNAs) are small non-coding RNAs that regulate gene expression during animal development. Large scale efforts to identify functions of individual miRNAs or miRNA families provide invaluable insight into the roles these miRNAs play in organism development (Miska *et al.* 2007, Alvarez-Saavedra and Horvitz 2010), however, some miRNA genes lack deletion alleles. To our knowledge, no alleles in *C. elegans mir-1022* gene are currently available.

Using CRISPR/Cas9 genome editing technique, we generated two novel alleles in the *mir-1022* locus in *C. elegans* (Fig 1A)*.* The *zen97* allele is a 498 pair deletion of the *mir-1022* locus which also inserts GAGGGGATT into that locus (Fig 1B). The *zen98* allele is a 508 base pair deletion from the *mir-1022* locus, inserting CGGTGTACAATGTATACAATGT in that location (Fig 1B).

To specifically target the *mir-1022* locus for editing, we used the following guide RNAs: *mir-1022*_gRNA 1: 5’-TCGGGCCAACACATTTCAG-3′ and *mir-1022_*gRNA 2: 5’-TTTGCGTGCGAAAGTGGTGA-3′. The primers used for PCR genotyping were mir-1022.for3: 5’-CTAGTGCATTGTCCAGGCAG-3′ and mir-1022.rev3: 5’-CGTGATCTCTTGGTGCACAT-3’. The Co-CRISPR marker *dpy-10* was used in order to more easily identify worms with active CRISPR/Cas9 as previously described (Arribere *et al.* 2014). CRISPR components were injected into N2 animals as an RNP complex (Paix *et al.* 2015). Alt-R Cas9 (cat# 1081058), *mir-1022* and *dpy-10* Alt-R® CRISPR-Cas9 crRNAs (custom), and tracer RNA (cat# 1072532) were purchased from IDT. PCR screening identified two independent mutations, each disrupting the *mir-1022* locus (Fig 1), which were subsequently homozygosed and sequenced. UY262 *mir-1022(zen97)* did not segregate any dumpy, dumpy roller, or roller animals and was not outcrossed. *mir-1022(zen98)* was outcrossed once to wild type (N2) males to remove a background *dpy-10* mutation, generating the 1x outcrossed UY286 *mir-1022(zen98)* strain. Sequencing was repeated to confirm the mutation.

Both alleles are homozygous viable. While not extensively characterized, no gross morphological phenotype was produced by either *mir-1022* allele. Further phenotypic analysis will be necessary to determine the exact effect of the two *mir-1022* mutations.

## Reagents

UY262 *mir-1022(zen97)* and UY286 *mir-1022*(*zen98)* (1x outcrossed) are available upon request.
